# Grappling With the COVID-19 Health Crisis: Content Analysis of Communication Strategies and Their Effects on Public Engagement on Social Media

**DOI:** 10.2196/21360

**Published:** 2020-08-24

**Authors:** Cindy Sing Bik Ngai, Rita Gill Singh, Wenze Lu, Alex Chun Koon

**Affiliations:** 1 The Department of Chinese and Bilingual Studies The Hong Kong Polytechnic University Hung Hom, Kowloon Hong Kong Hong Kong (China); 2 The Language Centre Hong Kong Baptist University Hong Kong Hong Kong (China); 3 School of Biomedical Sciences Li Ka Shing Faculty of Medicine The University of Hong Kong Hong Kong Hong Kong (China)

**Keywords:** COVID-19, communication, public engagement, social media, infodemiology, infodemic, message style, health content frames, interactive features, framework, content analysis

## Abstract

**Background:**

The coronavirus disease (COVID-19) has posed an unprecedented challenge to governments worldwide. Effective government communication of COVID-19 information with the public is of crucial importance.

**Objective:**

We investigate how the most-read state-owned newspaper in China, People’s Daily, used an online social networking site, Sina Weibo, to communicate about COVID-19 and whether this could engage the public. The objective of this study is to develop an integrated framework to examine the content, message style, and interactive features of COVID-19–related posts and determine their effects on public engagement in the largest social media network in China.

**Methods:**

Content analysis was employed to scrutinize 608 COVID-19 posts, and coding was performed on three main dimensions: content, message style, and interactive features. The content dimension was coded into six subdimensions: action, new evidence, reassurance, disease prevention, health care services, and uncertainty, and the style dimension was coded into the subdimensions of narrative and nonnarrative. As for interactive features, they were coded into links to external sources, use of hashtags, use of questions to solicit feedback, and use of multimedia. Public engagement was measured in the form of the number of shares, comments, and likes on the People’s Daily’s Sina Weibo account from January 20, 2020, to March 11, 2020, to reveal the association between different levels of public engagement and communication strategies. A one-way analysis of variance followed by a post-hoc Tukey test and negative binomial regression analysis were employed to generate the results.

**Results:**

We found that although the content frames of action, new evidence, and reassurance delivered in a nonnarrative style were predominant in COVID-19 communication by the government, posts related to new evidence and a nonnarrative style were strong negative predictors of the number of shares. In terms of generating a high number of shares, it was found that disease prevention posts delivered in a narrative style were able to achieve this purpose. Additionally, an interaction effect was found between content and style. The use of a narrative style in disease prevention posts had a significant positive effect on generating comments and likes by the Chinese public, while links to external sources fostered sharing.

**Conclusions:**

These results have implications for governments, health organizations, medical professionals, the media, and researchers on their epidemic communication to engage the public. Selecting suitable communication strategies may foster active liking and sharing of posts on social media, which in turn, might raise the public’s awareness of COVID-19 and motivate them to take preventive measures. The sharing of COVID-19 posts is particularly important because this action can reach out to a large audience, potentially helping to contain the spread of the virus.

## Introduction

### Background

The first known coronavirus disease (COVID-19) case was reported in China on November 17, 2019 [1], and on January 23, 2020, the government in China imposed a strict lockdown in Wuhan, the epicenter of the virus. Despite a massive containment effort, by late February, 80,000 cases had emerged [2]. By March, COVID-19 was confirmed in many countries worldwide and the World Health Organization (WHO) declared COVID-19 a pandemic on March 11, 2020 [3,4]. Pandemics in the past such as the 2003 severe acute respiratory syndrome and H1N1 have had significant impacts on people’s lives, socioeconomic activities, and population movement [5]. COVID-19 also presented similar impacts, but its spread was even faster [6]. A pandemic requires large-scale immediate actions by the government to connect with the public and a change in behavior of the public to combat the rapid spread of the disease [7]. For a new disease such as COVID-19, effective epidemic communication is crucial to inform the public about the latest updates of the disease, motivate them to adopt preventive measures to minimize the transmission of the disease, and reassure them that the government is capable of handling the situation [8-11]. Many studies on epidemic and pandemic communication exist on traditional media [8,12,13], suggesting that the public learns about the health risks associated with the pandemic from the media [14,15], which affects how they respond to the epidemic or pandemic [16]. In recent years, social media has played an increasingly important role in promoting health risk communication during an epidemic [17,18]. Research on the use of social media to investigate public attention to new epidemics has been conducted, such as with H7N9 [19-21], the Ebola outbreak [9], and the H1N1 pandemic in 2009 [22]. However, there are few studies that have adopted social media analysis in examining government media communication with the public and the public’s response to the new COVID-19 epidemic [10,11]. Because timely public action is needed to contain the spread of the new disease, it is of urgent importance to investigate how the government media communication engages the public. This information can provide insights on what the media, health organizations, and government can further do to disseminate information to the public so that the latter can take appropriate measures to stem the spread of the virus.

In terms of what organizations emphasize in their epidemic or pandemic communication, a prior study [22] found that most corporate and government organizations in the United States relied on the content frames of health crises, health issues, and disasters in communicating messages about the 2009 H1N1 flu pandemic with the public. Government organizations were more likely than corporate organizations to frame the H1N1 pandemic as a general health issue and emphasized uncertainty, disease detection, and preventive measures [22]. The style of communication can also have an impact on public engagement in that a narrative style has a positive effect on preventive and detection health behaviors [23] and arguments and facts may be used too [23]. Researchers have pointed out that narratives promote health behavior change [24,25], yet there is a lack of research on the use of narratives in pandemics for effective health communication, apart from Sandell et al [13] who revealed that positive narratives were effective in raising the public’s awareness of health risks and the preventive measures to curb the spread of the 2009 H1N1 pandemic [13]. Additionally, interactions on social media can affect health behavior and attitudes [26], and thus, the creation of the dialogic loop via the use of interactive features [27,28] is important on social media. This can be done by allowing the public to post questions and receive feedback [27,29] and using interactive features such as multimedia and hashtags [30]. A previous study has found a positive relationship between chief executive officers’ (CEOs) use of hashtags and public engagement with respect to likes, shares, and comments on social media [28]. However, a research gap exists in understanding the interactive features used by the government in its communication with the public on social media with regard to a pandemic.

Synthesizing this literature, our study was guided by the observation that there is scant research on the use of social media to disseminate information about COVID-19 and public engagement with this information [10,11]. In particular, there is a research gap in understanding the content frames employed by the government’s media in the Chinese context, its style of communication, and the use of interactive features in its communication with the public regarding a new epidemic. Therefore, in this study, we investigated how the most-read government-owned newspaper in China, *People’s Daily*, serving as the main vehicle for the government’s dissemination of information to the public, employed a social networking site, Sina Weibo, to communicate and possibly engage the public with COVID-19.

As of 2014, China had 649 million internet users [31]. To use the power of the internet, the main Chinese state-owned media such as *People’s Daily* and *CCTV News* have shifted the paradigm of media coverage by placing more emphasis on communication with the public via social media [32]. They have also switched to a more interactive style to better connect with the public [32]. In China, where Facebook is blocked, Weibo, a social media platform under *People’s Daily* introduced by the Chinese commercial corporation Sina, has taken over and become the largest social media network [33]. In 2018, Weibo had over 462 million active users [34] and was used by approximately 200 million people every day [35].

With years of the government’s continued efforts, the reputation of the Chinese state-owned media has improved significantly in the eyes of the Chinese public [36]. State-owned media such as *People’s Daily* now maintain a strong web presence and a user-friendly image rather than an authoritative image [37]. *People’s Daily* encourages its audience to participate in discussions and demonstrates a strong tendency to adopt positive and persuasive messages [38]. For example, on a topic of haze-related issues, instead of providing pictures of haze with a negative valence, *People’s Daily* posted positive images that encouraged the public to appreciate the beauty of nature, accompanied by persuasive messages that suggested substantial improvements to be made in the future [38]. This is vastly different from how *China Daily* handled the same topic, which displayed a cartoon of Santa Claus hitting a tree due to haze [38]. This example demonstrates that the state-owned media in China*, People’s Daily*, and its online platform, Sina Weibo, have actively adapted their styles of interactive communication to better engage the public.

In terms of health emergency communication, previous studies have found that social media platforms such as Twitter and even the photograph-based Instagram played a significant role in guiding the public during the Zika virus outbreak in 2016 [18,39]. For China, Sina Weibo performs a similar role during pandemics since the government, news media, and the public heavily relied on it as an online platform for communicating information during the current COVID-19 outbreak [11]. Sina Weibo serves as a pivotal communication platform for the government to interact with the public and disseminate information about COVID-19, such as its symptoms, preventive measures, and adopted health policies [11]. Therefore, we contend that *People’s Daily* would also communicate information about COVID-19 and interact with the public on its social media platform, Sina Weibo. In our study, we integrated factors, including health crises framing in the media context [22,40-42], message style in health communication [13,23], and interactive features [27,28] to examine epidemic communication and public engagement in China. We then developed an integrated framework to investigate the relationship between these factors and the levels of public engagement. Since our study also investigated public engagement in the form of likes, comments, and shares, it might offer insights on how effectively social media platforms such as Weibo can be used for epidemic communication.

### Developing an Integrated Framework

The WHO has advised governments to take proactive steps to communicate with the public about epidemics, as the sharing of critical information about the epidemic can minimize the spread of the disease and foster the public’s collaboration with the government [10,43,44]. Social media serves as a major communication platform for the government and public health authorities to provide timely health information to the public [11,22,45-47]. The contribution of this study is that we incorporated three key dimensions in health emergency communication on social media, namely, the framing of health crises and issues [22,40-42], message style [13,23], and the interactive loop [27,28] to examine COVID-19 communication by the government-owned media and public engagement in China. Our findings shed light on how responses to the epidemic are framed by the media and what encourages the public to engage with such communication and take appropriate actions to slow the spread of the virus. In the following, we explain the three dimensions adopted in our study: content frame, message style, and interactive features.

#### Content Frame Dimension

Communication related to health risks depends on persuasion for the framing of the message that informs the public about important information and motivates them to act [48]. Framing refers to how a text or message defines an issue and provides the necessary context [49,50]. Entman [51] pointed out that “to frame is to select some aspects of a perceived reality and make them more salient in a communicating text.” Drawing on framing analysis, one can identify how organizations and the government frame their messages pertaining to critical issues for the public [52], thereby impacting the effectiveness of the information disseminated [53].

In the management of a health crisis, the media and government tend to employ six frames in message delivery: conflict (aspects of crises that bring tensions between parties), action (past or current crisis response actions), consequence (the effects or severity of the crisis), new evidence (discovery of new evidence that contributes to the crisis understanding), uncertainty (aspects such as the spread of the epidemic, treatment, and what is unknown), and reassurance (reassuring the public) [22,41]. When handling communication of health issues, five frames in the delivery of health messages are noted, namely, disease detection (symptoms to indicate how the disease is spreading), disease prevention (taking preventive measures), health care services (the actions that the health care system is taking), scientific advances (discovery of new evidence showing how the disease is spread), and lifestyle risk factors (personal habits that are likely to lead to the disease) [22,40,42]. In the application of these frames, Liu and Kim [22] noted that most corporate and government organizations in the United States used the frames of health crises and health issues much more via traditional media than social media in disseminating messages about the 2009 H1N1 flu pandemic [22]. Yet corporate organizations framed the pandemic as a health crisis rather than as a general health issue, meaning that they did not emphasize the long-term actions that could prevent the health issue from arising in future. In addition to this, Liu and Kim [22] noted that government organizations were more likely to use uncertainty subsumed under the health crisis frame whereas corporate organizations tended to use the conflict indicator [22]. In another study, Shih et al [41] noted that the frames of governmental action and consequence were predominantly used by journalists to craft stories about epidemics including mad cow disease, West Nile virus, and avian flu in the print version of New York Times [41].

Given that COVID-19 was a health crisis and health issue emerging in China and required immediate action from the public, we contend that framing this epidemic using the health crisis frames of action, new evidence, uncertainty, and reassurance, would be of relevance to communication with the public, while the frame of health issues, namely, disease prevention and health care services, are of salient importance too since information is lacking on the details and duration of the epidemic. As highlighted by Shih et al [41], the government may attempt to minimize loss by reassuring the public with actions and new evidence via its influence on the media and its frames. Therefore, the previously mentioned frames could be effectively used in the media coverage of the epidemic. For a new epidemic, vaccines and medicine are not available to the public, so disease detection and scientific advances are tasks that only medical professionals can undertake and, thus, may not be able to engage the public. Disease prevention is vital and includes information about what preventive measures the public should adopt to reduce the risk of infection [12,43]. A prior study [22] found that government organizations in the United States were more likely to incorporate uncertainty into their crisis responses to the H1N1 pandemic, and with the implications of their results, we incorporated uncertainty into our framework, since the newspaper we examined is the main vehicle used by the government in China to communicate with the public. Uncertainty is useful because by indicating what is unknown, more transparency of information is provided, possibly generating trust [14,43]. Conflict was primarily used by corporate organizations as opposed to government organizations for the H1N1 pandemic in the United States [22] and, thus, not deemed of specific value in our framework.

The frames that we employed are in line with the information that the WHO recommends that the media should provide to the public: offering accurate and transparent information to the public; encouraging appropriate attitudes, actions, and behaviors; and helping prevent unnecessary fear [44]. As a result, we combined the eleven frames of health crises and issues into six frames for the investigation of COVID-19 content frames in social media posts, namely, *(1) action*, *(2) new evidence*, *(3) reassurance*, *(4) disease prevention*, *(5) health care services,* and *(6) uncertainty*. Since these frames are all content-related, we termed them *subdimensions* under the *content frame* dimension.

#### Message Style Dimension

Since a key objective of epidemic communication is to persuade the public to change their behavior to limit the spread of the disease [11] while the public has a need for real-time information [47], effective messages need to be designed, requiring some form of appeal. In this regard, the effectiveness of narratives in health communication on disease detection and prevention has been explored [54-56]. Narratives refer to stories that people use and tell, and consist of anecdotes and personal stories with plots [23,24]. Narratives engage the public because they make them concentrate on the story events instead of disputing the presented information while eliciting emotional reactions and being both entertaining and informative [23,46,55,57,58]. On the other hand, nonnarrative messages depend on the use of arguments and facts presented logically and are considered as informative [23].

Studies on the effectiveness of narratives in brand advertisement connection with customers and in the area of health communication have been conducted [58-60]. For example, a narrative film was effectively employed to communicate the need for vaccination against the human papillomavirus [25]. Scholars have increasingly recognized the role of narratives in promoting health behavior change [24], but studies on the use of narratives in pandemics for effective health communication are scarce with the notable exception of Sandell et al [13], who found that positive narratives were powerful in raising the public’s awareness of health risks and preventive measures for the 2009 H1N1 pandemic [13]. Based on this, we categorized *narrative* and *nonnarrative* as subdimensions under the *message style* dimension.

#### Interactive Features Dimension

The interaction (ie, one-to-one or one-to-many) on social media sites can influence health behavior and attitudes [26], and consequently, the promotion of the dialogic loop with interactive features [27,28] is crucial on such sites. An interactive dialogic loop allows the public to post questions and receive feedback as well as post comments and share them [20,27]. A wide range of interactive features are available on sites, including multimedia (eg, videos, audio, photos, podcasts), stay-up-to-date tools such as hashtags, and comments on content [30]. Hashtags enable users to find relevant shares on an issue [61] and facilitate in making synchronous conversations on Twitter, thereby fostering engagement [62], with a study noting a positive relationship between CEOs’ use of hashtags and engagement in terms of likes, shares, and comments [28]. To encourage users to return to the site, an attractive site and relevant links are necessary. Regarding conservation of visitors, the site should include useful external links [27]. In health-related communication, it is known that social media posts with interactive features leave a deep impression on the public when compared with posts in plain text [63]. Hence, we assigned “interactive features” as the third dimension, comprising the four subdimensions of *(1) links to external sources*, *(2) use of hashtags*, *(3) use of questions to solicit feedback*, and *(4) use of multimedia*.

Although prior studies have recognized the importance of the content frames [22,40-42], message style [13,23], and interactive features [27,28] in health-related communication, the question as to whether these three dimensions can facilitate the communication of COVID-19 on the government’s social media platform remains unclear. Therefore, our first research question (RQ) is derived:

- RQ1: How frequently did the official social media employ the subdimensions of content frames, message style, and interactive features in its communication of COVID-19?

A clearer indication of the public’s awareness of the information communicated by the government can be revealed through their actions of liking, sharing, and commenting on the government’s posts. Therefore, it is pertinent to investigate the effects of the content frames, message style, and interactive features on different levels of public engagement [62,64,65]. Social media users may use “likes” to indicate their interest in a health issue [66,67], and by commenting and sharing, the public can let others know that the issue is important, thereby serving as disseminators of the original message posted [9]. To investigate differences in public engagement with health information posted by the government in response to COVID-19, our second RQ is posed:

- RQ2: Did the subdimensions of content frames, message style, and interactive features have different levels of impact on public engagement?

Different dimensions may function synergistically to impact public engagement. As has been found in a study, an interaction effect between content and style of communication on public engagement in brand social media communication was observed [68]. It is, therefore, likely that interaction effects might exist between some of the dimensions or subdimensions on public engagement in COVID-19 communication. Thus, our third RQ is as follows:

- RQ3: Could the dimensions (ie, content frames, style, and interactive features) or subdimensions interact synergistically to increase or decrease the levels of public engagement with the government’s communication of COVID-19?

By examining the impact of content frames, message style, and interactive features on public engagement in COVID-19 communication, our study aims to provide meaningful and critical information for governments, health organizations, communication professionals, and researchers regarding the health emergency communication strategies employed and their effectiveness in raising the public’s awareness of and urgent need for taking preventive measures against COVID-19.

## Methods

### Data Collection

We selected the government-owned social media platform *People’s Daily*’s Sina Weibo account for data collection. *People’s Daily* is the official newspaper of the Central Committee of the Communist Party of China [69] for disseminating government information to the Chinese public [70]. It is the most influential and authoritative newspaper in China, having a circulation of 3 million, and is ranked as one of the world’s top 10 newspapers [71]. With 117 million followers, Sina Weibo of *People’s Daily* is also one of the top followed and most visited Sina Weibo sites in China. Due to the prominent use of Sina Weibo for social media communication in China [34] with 462 million active online users in 2018, we captured all posts and the public’s responses communicated between the government and public on COVID-19 from *People’s Daily* for the investigation of government communication of COVID-19 and its interaction with the public.

### Sample Period

A text corpus containing all posts on Sina Weibo of *People’s Daily* pertaining to COVID-19 from January 20, 2020, to March 11, 2020, was constructed. The sampled period began on January 20, 2020, when the Chinese State Council officially announced the management of COVID-19 as a public health emergency issue and the corresponding preventive measures were launched to tackle COVID-19 [72,73]. The sampled period ended on March 11, 2020, when the WHO declared the COVID-19 outbreak a pandemic, meaning that the regional epidemic had become a global public health emergency [4]. Subsequently, all online posts related to COVID-19 were manually extracted from Sina Weibo’s account of *People’s Daily*, and in total, 3255 posts were collected.

### Sample Size and Sample Data Collection

To generalize a sample size to represent the target population (3255 posts), we employed the sample size calculator developed by the Australian Statistics Bureau to estimate a sample size of 620, giving a confidence level of 95%, a confident interval of 0.035, and a standard error of 0.018. A random sampling method was employed. The 620 posts and their corresponding public responses (ie, number of shares, comments, and likes) on *People’s Daily*’s Sina Weibo account from January 20, 2020, to March 11, 2020, were harnessed for quantitative content analysis. To systematically detect statistically valid outliers, we employed z score to quantify the unusualness in the observations [74]. There were 12 posts (2%) identified as outliers and removed from the data pool. These outliers included posts that were significantly longer or shorter, which would have otherwise caused problems during content analysis, as the length of the posts would affect the number of counts in content themes, style, and interactive features. Consequently, 608 posts and the related public responses were included in the corpus for content analysis.

### Content Analysis and Coding Scheme

Content analysis was employed to examine COVID-19 communication in the 608 posts of *People’s Daily*’s Sina Weibo. Content analysis is a widely employed method in the study of technical and media communication [75]. It is concerned with the context in which the occurrences of words, phrases, signs, and sentences are recorded and analyzed to provide an in-depth understanding [75]. Researchers can design a variety of categories based on their interactions with the data to develop an integrated framework for quantitative studies [76]. Content analysis can be applied to “virtually any form of linguistic communication to answer the classic questions of who says what to whom, why, how, and with what effect” [77]. Therefore, it is well-suited to a coding operation involving a developed framework in the media communication context [78]. Through an in-depth analysis of mainstream media communication, we were able to reveal and establish the relationship between the variables in the proposed conceptual framework.

First, to answer RQ1, we drew on previous studies of epidemic communication, health crisis communication, and public relations studies [11,40] to code the topics of the content dimension exhibited in the government’s COVID-19 communication into the following six subdimensions on a sentence basis: *(1) action*, *(2) new evidence*, *(3) reassurance*, *(4) disease prevention*, *(5) health care services*, and *(6) uncertainty*. Second, to examine the communication styles of COVID-19 posts from *People’s Daily*, we built on prior studies [55,58] and coded the two message styles in the style dimension into the subdimensions of *(1) narrative* and *(2) nonnarrative* on a sentence basis. To determine whether the narrative style of communication was employed, we examined if the post had a temporal or spatial sequence and revealed the writer’s feelings or thoughts. Last, we built on prior public relations studies [27,38,68] and coded the number of interactive features used to facilitate the creation of the interactive dialogic loop. These interactive features included *(1) links to external sources*, *(2) use of hashtags*, *(3)* *use of questions to solicit feedback*, and *(4) use of multimedia* (see Multimedia Appendix 1 for the exemplifications of coding items and examples extracted from the collected posts).

Regarding RQ2 and 3, we recoded the dimensions of content, style, and interactive loop using the dominant category for performing the analysis of variance (ANOVA) tests on content, style, and interactive loop on public engagement. For example, we found 43% of the sentences in post number 128 belonging to *action* and 29% to *disease prevention*; 57% of sentences employed a narrative style of communication while 43% were nonnarrative; 1 link provided an external source, 2 pairs of hashtags, and 1 multimedia feature. We then recorded the content as *action* based on the dominant content topic, style as *narrative* based on the dominant use of narrative sentences, and interactive loop as *use of hashtags* based on the dominant use of hashtags. If the count of sentences or interactive features was the same, the primary coder checked the title, topic sentences, and context of the post to determine the dominant category.

To address RQ2 and 3, we recorded the number of shares, comments, and likes of the sampled posts to investigate the relationship between *People’s Daily* communication and its impact on public engagement. Regarding the negative binomial regression (NB2) analysis, the coding results of RQ1 were adopted to investigate the effect of all subdimensions on public engagement. The number of shares, comments, and likes in RQ2 and 3 were also included for statistical analyses.

### Intercoder Reliability

The coding was conducted by the third author (the primary coder) and a well-trained coder who all possess a postgraduate degree in communication. To ensure intercoder reliability on the coding of dimensions, subdimensions, and public engagement, the coder was repeatedly trained on the coding scheme. Any disagreement between the author and coder was discussed in the coding process. The measure of intercoder reliability was based on the co-coding of 120 posts from the data pool of 608 posts (19% of the total number of posts sampled) [75]. For all categories, the average agreement was higher than 0.83, and the average Cohen kappa was greater than 0.8, indicating an almost perfect agreement [76] (see Multimedia Appendix 2 for intercoder checking results of all categories).

### Statistical Analyses

To analyze the differences in the frequencies of the use of each subdimension in the communication of COVID-19–related news by the official social media (RQ1), we coded the presence of subdimensions in each of the 608 posts and then calculated the mean counts for each of the 12 subdimensions. We then employed the one-way ANOVA and the post-hoc Tukey test in SPSS (IBM Corp) to reveal the differences in the use of content, style, and interactive features in COVID-19 social media communication (RQ1) and the difference in the number of shares, comments, and likes in relation to the subdimensions of content, style, and interactive dimensions (RQ2). The two-way ANOVA was performed to examine the interaction effect of content and style on public engagement in the form of shares, comments, and likes (RQ3).

To test the assumptions of normality in ANOVA, we performed the Kolmogorov-Smirnov and Shapiro-Wilk tests on the normality of the variables. Most variables were not normally distributed, but we decided to continue using ANOVA as it has proven to be robust and valid in testing the difference between independent variables, even if the normality assumption is violated [77]. In addition, we conducted the test of homogeneity of variances when performing ANOVA. When the assumption of homogeneity of variances was violated, the ANOVA results were replaced with those of the Welch ANOVA.

As for RQ2, which involved examining the relationship between the 12 subdimensions (independent variables) and the public’s responses in terms of the count number of shares, comments, and likes (dependent variables), we first employed Poisson regression, a count regression model in SPSS [78,79]. However, real-world data sets are commonly known to violate the assumption in the Poisson regression with respect to overdispersion of outcome variables [80]. As expected, such a violation was detected in our data set, and thus, we followed the common practice of replacing Poisson regression with the NB2 [80] to improve the goodness of fit, especially Akaike information criterion and bayesian information criterion. NB2 is effective in fitting various types of data arising in technical and communication research [81], and NB2 is a more general model that relaxes the strong assumption that the underlying rate of the outcome is the same for each included participant [81].

## Results

**Figure 1:**
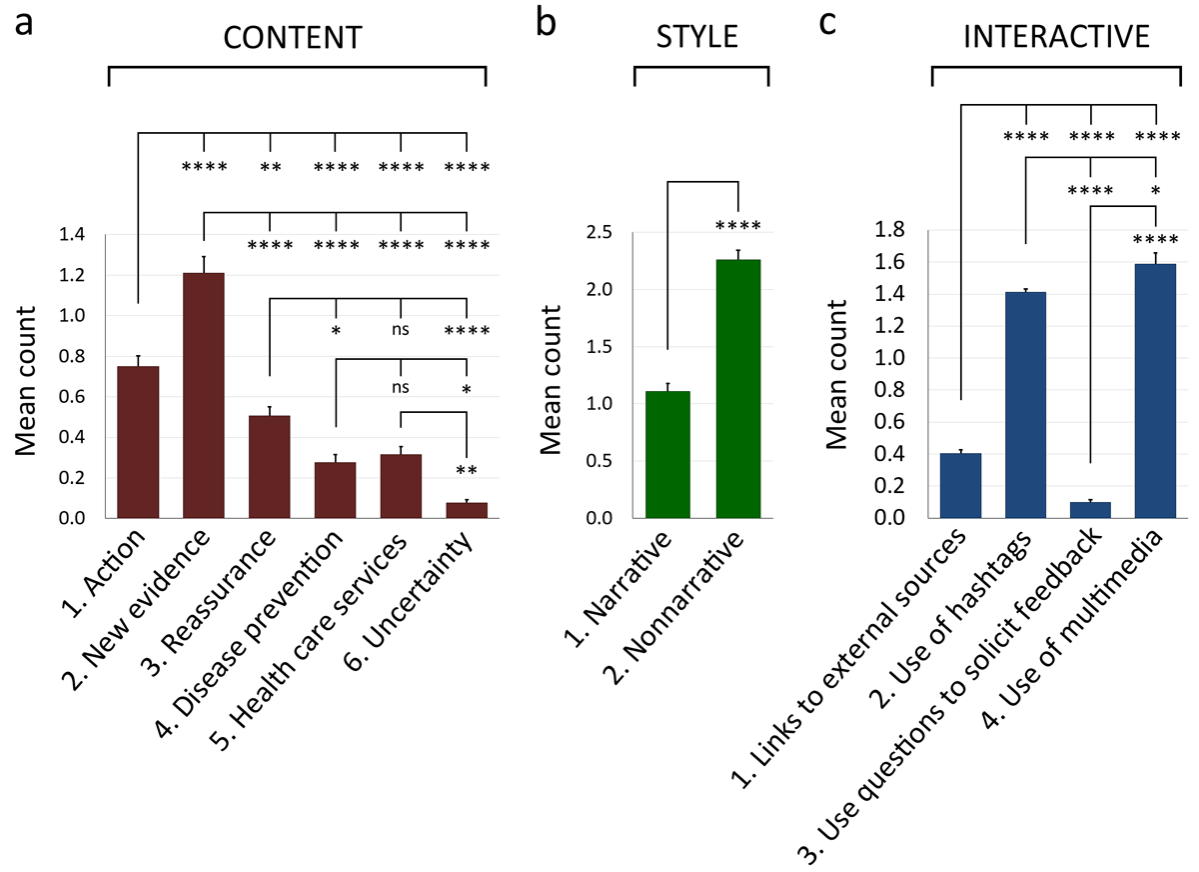
Comparison of the mean counts of subdimensions within each of the three dimensions. (a) Mean counts of subdimensions under the content dimension. (b) Mean counts of subdimensions under the style dimension. (c) Mean counts of subdimensions under the interactive dimension. *P<.05, **P<.01, ***P<.001, ****P<.0001. All histograms depict mean and standard error of the mean.

In response to RQ1 regarding the differences in the frequencies of each subdimension’s use in the communication of COVID-19–related news by the official social media, we found that *new evidence* in the content dimension was the most used subdimension (mean 0.749, standard error of the mean [SEM] 0.05) and significantly used much more than any other subdimensions (Figure 1a). *Action* was the second most prevalent subdimension (mean 1.210, SEM 0.08), and *reassurance* was the third most frequently used one (mean 0.506, SEM 0.05). *Disease prevention* (mean 0.276, SEM 0.04) and *health care services* (mean 0.315, SEM 0.04) ranked fourth and fifth respectively, and *uncertainty* was the least used subdimension (mean 0.077, SEM 0.02; Figure 1a). In relation to the style dimension, the *nonnarrative* (mean 2.259, SEM 0.09) style was used approximately twice as much as the *narrative* (mean 1.110, SEM 0.07) one (Figure 1b). Concerning the interactive dimension, the *use of multimedia* (mean 1.586, SEM 0.08) and *use of hashtags* (mean 1.411, SEM 0.02) were the most prevalent subdimensions, with the *use of multimedia* being slightly but significantly higher than that of the *use of hashtags* (Figure 1c). By contrast, both *links to external sources* (mean 0.402, SEM 0.02) and * use of questions to solicit feedback* (mean 0.097, SEM 0.02) were used infrequently, with the * use of questions to solicit feedback* being used significantly less in comparison to all other subdimensions (Figure 1c).

**Figure 2:**
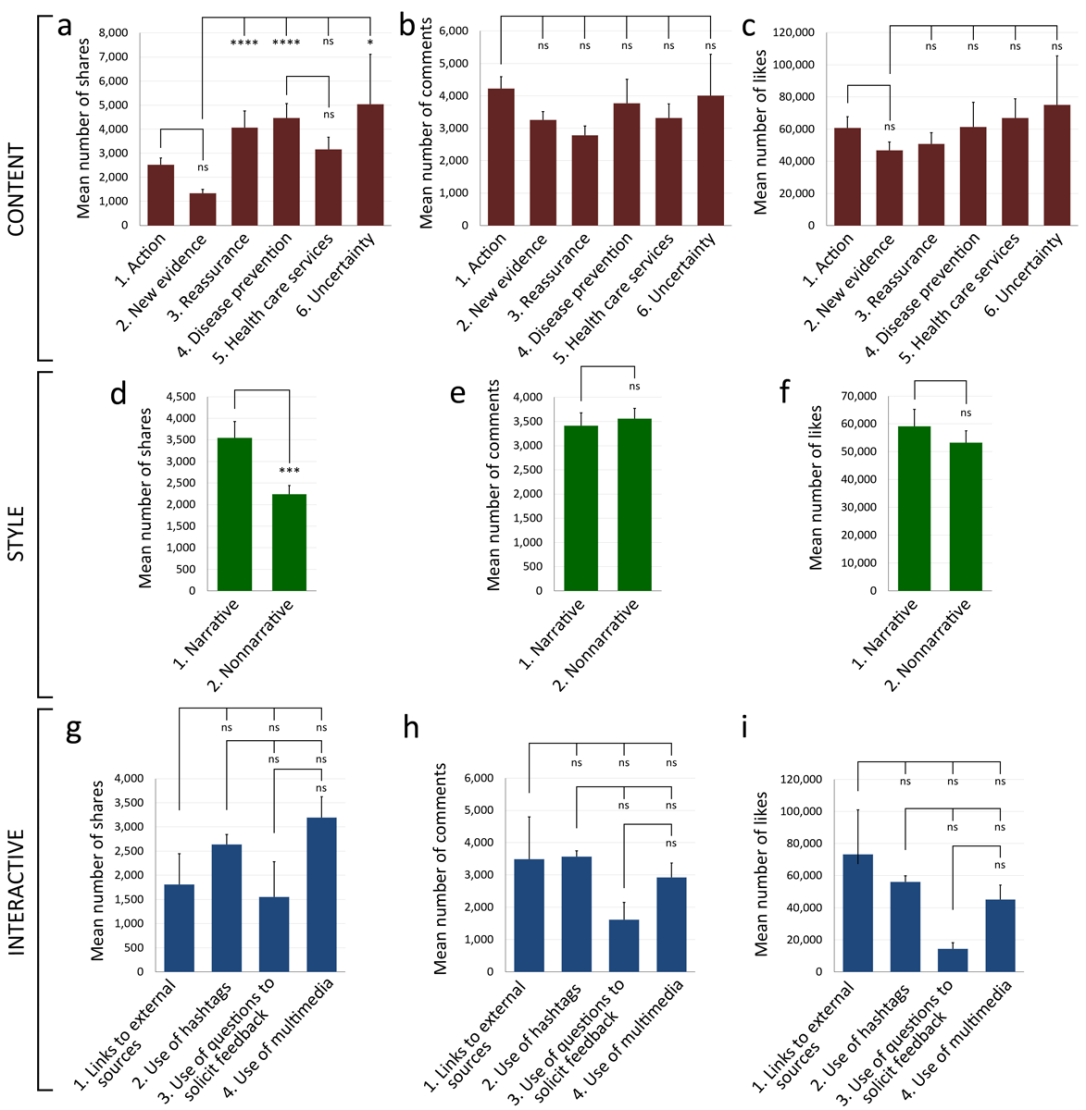
Comparison of the mean number of shares, comments, and likes for posts of each subdimension. Mean number of (a) shares, (b) comments, and (c) likes of the subdimensions of the content dimension. Mean number of (d) shares, (e) comments, and (f) likes of the subdimensions of the style dimension. Mean number of (g) shares, (h) comments, and (i) likes of the subdimensions of the interactive dimension. *P<.05, **P<.01, ***P<.001, ****P<.0001. All histograms depict mean and standard error of the mean.

Regarding the levels of impact on public engagement from individual subdimensions in COVID-19 social media posts (RQ2), our results showed that posts of *new evidence* generated the least number of shares of all six subdimensions (mean 1327.81, SEM 165.90). Posts of *new evidence* had significantly fewer shares than posts of *reassurance* (mean 4065.32, SEM 689.88), *disease prevention* (mean 4455.71, SEM 604.95), and *uncertainty* (mean 5033.35, SEM 2242.13; Figure 2a). However, the six subdimensions of content did not show differences in their impact on comments and likes (Figure 2b, c). For the style dimension, *narrative* posts generated significantly more shares than *nonnarrative* posts (*narrative*: mean 3544.03, SEM 379.80 vs *nonnarrative*: mean 2237.06, SEM 204.18; Figure 2d). Similar to content, the message style did not exert any impact on the number of comments and likes (Figure 2e, f). As for the interactive dimension, no significant differences were observed among the four subdimensions in terms of shares, comments, and likes (Figure 2g-i). Surprisingly, although they were the most frequently used subdimensions (Figure 1a, b), *new evidence* and the *nonnarrative style* had the least impact on the number of shares in their own dimensions (Figure 2a, d).

To determine which of the twelve subdimensions were effective positive or negative predictors of public engagement in COVID-19 communication (RQ3), we fitted the share, comment, and like count data to a NB2 model. Our results in Figures 1 and 2 indicated that, although *new evidence* was the most used content subdimension (Figure 1a), its posts received the lowest number of shares (Figure 2a), suggesting a negative correlation between *new evidence* and the number of shares. In our NB2 analysis, we confirmed that *new evidence* was a strong negative predictor of the number of shares (β=–.253, SE 0.068, *P*<.001; Table 1). Similarly, *nonnarrative* was the most frequently used style (Figure 1b) but generated a lower number of shares as opposed to the *narrative* one (Figure 2d). Again, we confirmed that the *nonnarrative* style was indeed a strong negative predictor of the number of shares (β=–.223, SE 0.068, *P*<.001; Table 1). By contrast, the *narrative* style was found to be a strong positive predictor of the number of shares (β=.283, SE 0.064, *P*<.001; Table 1). For the interactive dimension, *links to external sources* was a strong positive predictor of the number of shares (β=.319, SE 0.087, *P*<.001), whereas the *use of multimedia* was a weak positive predictor of the number of shares (β=.057, SE 0.023, *P*=.02; Table 1). Finally, we noted that the * use of questions* *to solicit feedback* was a strong negative predictor of the number of comments (β=–.177, SE 0.087, *P*=.04) and likes (β=–.290, SE 0.111, *P*=.01; Table 1).

Subdimensions are likely to function synergistically in affecting public engagement. To examine whether there was an interaction among the dimensions on public engagement (RQ3), we performed a two-way ANOVA analysis on the mean number of shares, comments, and likes of the dimensions. Our results confirmed a significant interaction effect between content and style on the number of likes (Table 2). However, there was neither any interaction effect between content and the interactive dimension itself nor between style and the designated interactive dimension (Table 2).

**Table 1:** Identification of positive and negative predictors of the number of shares, comments, and likes using a negative binomial regression model.

Dimensions and subdimensions		Shares				Comments				Likes			
		β	SE	95% CI	*P* value	β	SE	95% CI	*P* value	β	SE	95% CI	*P* value
**Content**													
	Action	–.071	0.068	0.816-1.063	.29	.096	0.063	0.973-1.244	.13	.049	0.080	0.899-1.228	.53
	New evidence	–.253	0.064	0.685-0.881	*<.001* ^a^	.003	0.060	0.893-1.128	.95	–.053	0.077	0.816-1.102	.49
	Reassurance	–.053	0.072	0.824-1.092	.46	–.080	0.066	0.812-1.049	.22	–.059	0.086	0.797-1.116	.495
	Disease prevention	.057	0.078	0.909-1.234	.46	–.019	0.074	0.848-1.134	.79	–.054	0.097	0.783-1.145	.57
	Health care services	–.097	0.077	0.780-1.055	.21	.029	0.067	0.902-1.174	.67	.070	0.089	0.902-1.276	.43
	Uncertainty	–.090	0.138	0.697-1.197	.51	–.012	0.115	0.788-1.239	.92	.019	0.150	0.759-1.368	.90
**Style**													
	Narrative	.283	0.064	1.170-1.506	*<.001*	.069	0.055	0.961-1.194	.21	.129	0.074	0.984-1.316	.08
	Nonnarrative	–.223	0.068	1.094-1.427	*.001*	.037	0.061	0.921-1.169	.54	.108	0.078	0.955-1.299	.17
**Interactive**													
	Links to external sources	.319	0.087	1.160-1.633	*<* *.001*	.022	0.071	0.889-1.175	.76	.088	0.090	0.915-1.303	.33
	Use of hashtags	.079	0.081	0.923-1.268	.33	.059	0.070	0.925-1.216	.40	.016	0.092	0.848-1.217	.87
	Use of questions to solicit feedback	–.121	0.106	0.720-1.092	.26	–.321	0.090	0.608-0.865	*<.001*	–.463	0.116	0.501-0.790	*<.001*
	Use of multimedia	.057	0.023	1.011-1.108	*.02*	–.010	0.022	0.948-1.033	.64	–.046	0.029	0.903-1.011	.11

^a^Italics indicate a significant relationship.

**Table 2:** Test of interaction effect between content, style, and the interactive dimension.

Interactions			Shares											Comments											Likes								
			*df*		MS^a^		*F* test		*P* value		η_p_^2^		*Df*			MS		*F* test		*P* value		η_p_^2^		*df*			MS		*F* test		*P* value		η_p_^2^
**Content x style**																																	
	Content	5		74,362,796.0		3.627		.003		0.0295		5			13,679,259.3		0.804		.55		0.0067		5			1,600,144,996.5		0.213		.96		0.0018	
	Style	1		2,955,797.2		0.144		.70		0.0002		1			644,487.0		0.038		0.85		0.0001		1			856,248,211.1		0.114		.74		0.0002	
	Content x style	5		14,294,815.4		0.697		.63		0.0058		5			35,061,851.2		2.060		.07		0.0170		5			17,759,392,993.4		2.367		*.04* ^b^		0.0194	
	Error	597		20,504,947.6		N/A^c^		N/A		N/A		597			17,018,927.7		N/A		N/A		N/A		597			7,502,954,304.2		N/A		N/A		N/A	
**Content x interactive**																																	
	Content	5		14,588,523.9		0.706		.62		0.0059		5			7,622,337.7		0.452		.81		0.0038		5			2,892,590,073.7		0.384		.86		0.0032	
	Interactive	3		12,299,580.7		0.595		.61		0.0030		3			15,422,984.7		0.914		.43		0.0046		3			8,720,670,460.3		1.158		.33		0.0059	
	Content x interactive	9		7,690,008.8		0.372		.95		0.0056		9			12,698,088.8		0.753		.66		0.0114		9			6,061,808,678.1		0.805		.61		0.0121	
	Error	590		20,657,294.6		N/A		N/A		N/A		590			16,865,817.6		N/A		N/A		N/A		590			7,532,498,681.0		N/A		N/A		N/A	
**Style x interactive**																																	
	Style	1		4,166,367.8		0.195		.66		0.0003		1			543,790.1		0.032		.86		0.0001		1			454,343,258.8		0.060		.81		0.0001	
	Interactive	3		8,001,481.9		0.374		.77		0.0019		3			13,205,355.1		0.775		.51		0.0039		3			5,564,405,021.7		0.736		.53		0.0037	
	Style x interactive	3		11,004,271.3		0.514		.67		0.0026		3			4,937,789.1		0.290		83		0.0014		3			882,821,621.1		0.117		.95		0.0006	
	Error	600		21,411,044.7		N/A		N/A		N/A		600			17,039,291.1		N/A		N/A		N/A		600			7,560,858,471.6		N/A		N/A		N/A	

^a^MS: mean square.

^b^Italics indicate a significant interaction effect.

^c^N/A: not applicable.

**Figure 3:**
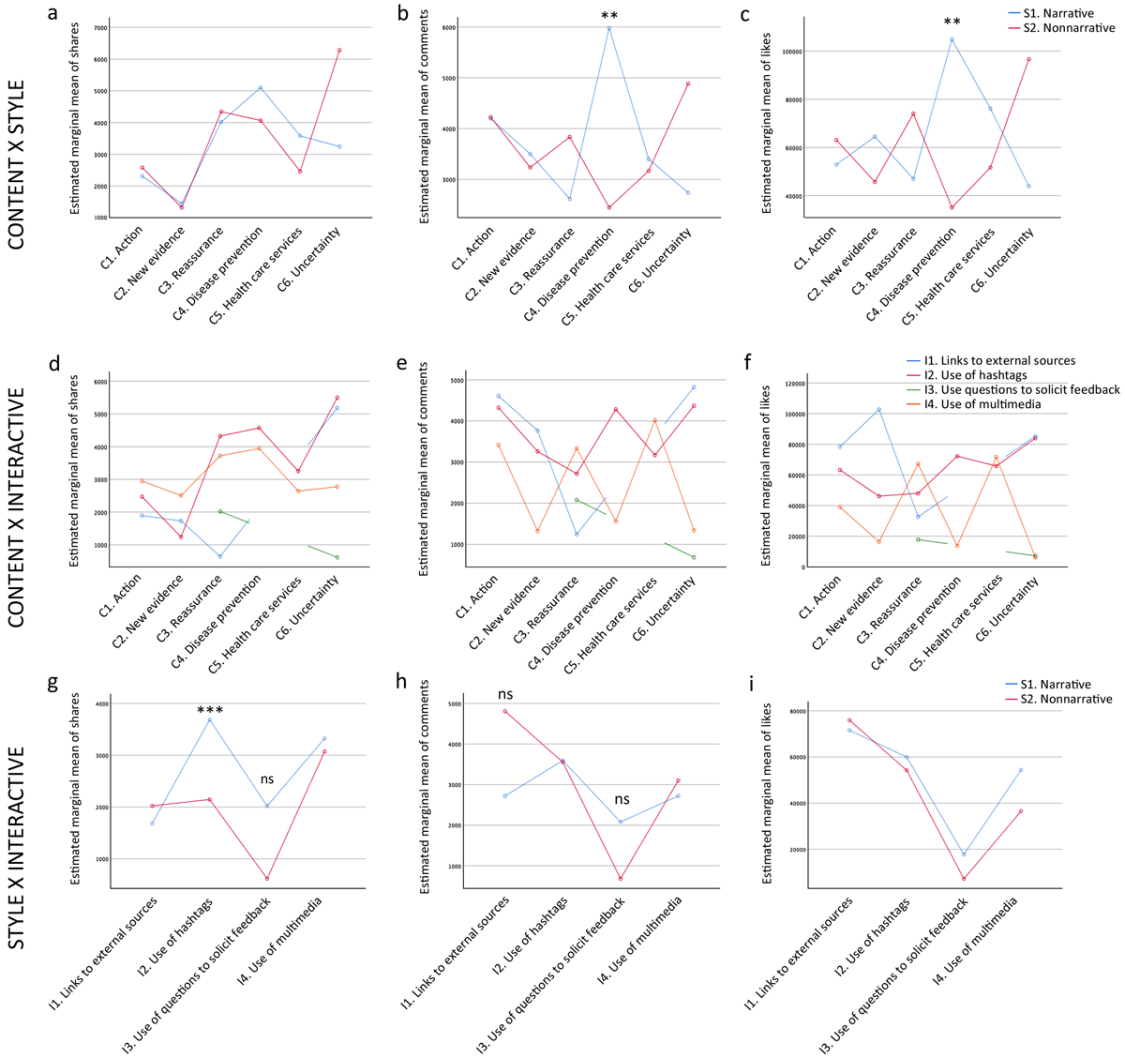
Simple main effects between the sub-dimensions on the number of shares, comments, and likes. Simple main effects between the sub-dimensions of content and style on the number of shares, comments, and likes (a-c). Simple main effects between the sub-dimensions of content and interactive loop on the number of shares, comments, and likes (d-f). Simple main effects between the sub-dimensions of style and interactive loop on the number of shares, comments, and likes (g-i). ** *P*<.01, *** *P*<.001.

To investigate the interactions between specific subdimensions, we performed simple main effect analyses to examine the interactions between specific subdimensions on the number of shares, comments, and likes. Between content and style, the different content subdimensions showed no significant differences in the number of shares between *narrative* and *nonnarrative* styles (Figure 3a). However, for the number of comments, the *narrative* style was significantly higher than that of *nonnarrative* in *disease prevention* posts (*narrative*: mean 5978.83, SEM 972.37 vs *nonnarrative:* mean 2446.33, SEM 753.19; *F*_1,597_=8.249, *P*=<.001, η_p_^2^=0.014; Figure 3b). Likewise, a higher number of likes was observed for the *narrative* style as opposed to *nonnarrative* in *disease prevention* posts (*narrative*: mean 104,881.00, SEM 20,416.43 vs *nonnarrative*: mean 35,092.87 SEM 15,814.50; *F*_1,597_=7.303, *P*=.01, η_p_^2^=0.012; Figure 3c). These results indicate that the pairing of *disease prevention* content with a *narrative* style generated a higher number of comments and likes.

Between the content and the interactive dimensions, no significant differences were observed in the number of shares, comments, or likes for the *narrative* and *nonnarrative* styles (Figure 3d-f). Between the style and the interactive dimensions, the *narrative* style received significantly more shares than the *nonnarrative* one on the *use of hashtag* posts (*narrative*: mean 3685.92, SEM 359.02 vs *nonnarrative*: mean 2145.31, SEM 245.16; *F*_1,601_=12.558, *P*<.001, η_p_^2^=0.021; Figure 3g), highlighting that the pairing of *narrative* style with the *use of hashtags* generated a higher number of shares. For the number of comments and likes, no significant differences were found (Figure 3h, i).

## Discussion

### Principal Results

Our results showed that a range of content frames, message styles, and interactive features was employed by the government to communicate about COVID-19 with the public on social media with a view to handling the health crisis. Yet different levels of engagement were revealed. In particular, *new evidence* and the *nonnarrative* style had the least impact on the number of shares (Figure 2a, d), although they were the most frequently used subdimensions (Figure 1a, b). Additionally, our NB2 results confirmed that *new evidence* and *nonnarrative* style were strong negative predictors of the number of shares (Table 2). On the other hand, the two-way ANOVA indicated that the pairing of *disease prevention* posts with a *narrative* style generated a higher number of comments and likes (Figure 3b, c), while the NB2 results confirmed that the *narrative* style was a strong positive predictor of the number of shares (Table 2). As found in an earlier study [47], posts on preventive and safety measures related to COVID-19 were most frequently employed by public health organizations in Singapore, the United States, and England, and our results on disease prevention posts were consistent with this study. In line with previous studies, our results also revealed the strong effect of the narrative style on public engagement [23,46,55,57,58]. A narrative style of communication fosters the public’s identification and emotional involvement through the character’s sharing in a story event [54,58]. Through this, health narratives can possibly raise the public’s awareness of health risks and encourage them to take actions to curb the spread of the disease [13,23,25,55,60].

A previous study has demonstrated an interaction effect between content and style [68], and therefore, we expected an interaction between these two dimensions. Indeed, our data showed a significant interaction between content and style on the number of likes (Table 2). With respect to the interaction between the subdimensions, our results showed that more shares were generated for posts related to *disease prevention, reassurance,* and *uncertainty* (Figure 2a) delivered in a *narrative* style (Figure 2d). *Links to external sources* and *use of multimedia* were also positive predictors of the number of shares (Table 2). A “share” indicates a high engagement level because it involves a cognitive action of disseminating the post to others, which can potentially reach a large audience [82-84]. Disease prevention is fundamental in a new epidemic [40-42] and uncertainty needs to be addressed because, by indicating what is unknown, more transparency of information is provided, thereby helping to build trust [14,43]. The public has a tendency to rely on social media during crises as the sites offer emotional support [85-87], indicating that the communication of uncertainty and reassurance might have served the purposes of offering emotional support and allaying anxiety. Our novel findings regarding the interaction between the subdimensions provide important insights for enhancing public engagement in epidemic communication on social media.

### Implications, Limitations, and Future Work

This study contributes to the understanding of what drives the public to be engaged with COVID-19 communication by the government and adds to the body of knowledge on public engagement with epidemic communication on social media. First, our integrated, comprehensive framework of public engagement with government health communication regarding COVID-19 in China was empirically tested. *People’s Daily* currently has 117 million followers, but Sina Weibo on its own is widely used for social media communication in China [34], with 462 million active online users in 2018. Existing followers of *People’s Daily* can influence other Weibo users through sharing the posts, fostering a sense of community with them, and potentially helping to contain the spread of COVID-19. Both “comments” and “likes” were noted for disease prevention posts delivered in a narrative style (Figure 3b, c). A “comment” is indicative of a high engagement level because it requires the user to read a post and respond to it [88] but the interpretation of a “like” is subject to change depending on the context. For example, one study suggested that a “like” is indicative of a lower engagement level [82], although within the context of epidemic communication, a “like” might be perceived as a user’s approval of the post’s importance. In view of this, both “likes” and “comments” are regarded as good indicators of health risk communication.

Second, *People’s Daily*’s approach of predominantly employing new evidence posts disseminated in a nonnarrative style in COVID-19 communication was not perceived as the ideal strategy to engage the public. We have gained insights into the subdimensions that can effectively enhance public engagement with epidemic communication; for instance, disease prevention posts delivered in a narrative style are viewed favorably. It is imperative for health organizations, governments, and researchers to use the public’s preferred subdimensions to increase the number of shares, comments, and likes with a view to effectively disseminating new epidemic information.

One of the limitations of this study pertains to the sampling period. Because we only captured the posts from a certain period of time, the results might vary in different time periods of an evolving epidemic. Our developed framework on COVID-19 communication with the public can be further empirically tested to assess the strength of the three dimensions and applied to other cultural contexts. As social media are frequently accessed by young people while there are demographics that still use traditional mass media in different ways, COVID-19 communication can be examined in terms of impact through other channels of behavior or practice too. An investigation into the use of other popular social media platforms such as WeChat in China to disseminate COVID-19 information can be conducted to gain more insights into this topic.

### Conclusions

In summary, this study presents a novel, comprehensive framework of the factors that engage the public in COVID-19 communication by the government on social media through empirically testing the measures of health content frames, style of messages, and interactive features. By drawing on this knowledge and harnessing the power of social media, governments and health organizations can determine which aspects to emphasize in an attempt to reduce the spread of the new disease.

## Appendix

Multimedia Appendix 1 Exemplifications of the content dimension, style dimension, interactive dialogic loop dimension, and relevant expressions identified from the corpus.

Multimedia Appendix 2 Summary of intercoder reliability statistics.

## Abbreviations

ANOVA: analysis of variance
CEO: chief executive officer
COVID-19: coronavirus disease 2019
NB2: negative binomial regression
RQ: research question
SEM: standard error of the mean
WHO: World Health Organization
