# Supplementary material for: Grappling With the COVID-19 Health Crisis: Content Analysis of Communication Strategies and Their Effects on Public Engagement on Social Media
Source: J Med Internet Res. 2020 Aug 24;22(8):e21360. doi: 10.2196/21360 (PMC7446717; doi:10.2196/21360)
Supplement: Multimedia Appendix 1 [file jmir_v22i8e21360_app1.docx]

**Appendix 1: Exemplifications of the content dimension, style dimension, interactive dialogic loop dimension and relevant expressions identified from the corpus**

| Coding items | Examples |  |
| --- | --- | --- |
| **Content dimension** | |  |
| 1. Action | No. 18: Jan.22, 15:16  #北京严查借疫情防疫之机哄抬物价行为#北京市场监管部门22日宣布已部署开展全面价格监管工作，严肃查处借疫情防疫之机囤积居奇、捏造散布涨价信息、哄抬物价等扰乱市场价格秩序的价格违法行为。欢迎广大市民积极拔打12345进行投诉举报，一经查实，监管部门将依法严肃处理，对情节恶劣的典型案件，将公开曝光。  Literal translation: #Beijing strictly prohibited product prices raising during the epidemic # Beijing Market Supervision Department announced on the 22nd that it had deployed comprehensive supervision on prices, seriously dealing with the hoarding of products, fabricated information, and spoofed prices as well as the illegal behavior which disrupted market price fluctuations. The public can call 12345 to make a complaint report. Once verified, the regulatory department will deal with it seriously according to the law, and the typical cases with serious circumstances will be publicly exposed. |  |
| 1. New evidence | No. 449: Feb.20, 17:01  【#中西医结合治疗新冠肺炎有效#】中医药局党组书记、副局长余艳红表示，在新冠肺炎疫情中坚持中西医结合取得了显著成效，大量临床实践证实，中西医结合治疗效果是肯定的，有效的。在重症预后方面有一个关键指标是淋巴细胞数量，研究发现#中医药可有效提高淋巴细胞数量#。  Literal translation: 【# The integration of Chinese and Western medicine is effective in treating COVID-19#】 Yu Yanhong, Secretary of the Party Leadership Group of the Chinese Medicine Bureau, said that adherence to the integration of Chinese and Western medicine has achieved remarkable results in the anti-COVID-19 situation. A large number of clinical practices have proved that the effect of integrated Chinese and Western medicine treatment is positive and effective. A key indicator is the number of lymphocytes. Studies have found that #TCM can effectively increase the number of lymphocytes #. |  |
| 1. Reassurance | No. 488: Feb.25, 11:15  【19位新冠肺炎重症患者集体出院，#李兰娟为出院患者颁战胜者证书#】李兰娟院士为武汉大学人民医院东院区19位新冠肺炎重症患者颁发“战胜者”证书。截至24日，该院区累计出院患者达118人。第100位出院患者是一位29岁的产妇。李兰娟院士为她颁发“战胜者”证书后，暖心地拉着她的手，关心她孩子的情况。  Literal translation: [19 patients were discharged collectively; #Li Lanjuan was awarded a “winner” certificate for discharged patients #] Academician Li Lanjuan issued “winner” certificates for 19 patients who were infected with COVID-19 in the Eastern Hospital of Wuhan University People's Hospital. Until February 24th, 118 patients had been discharged from the hospital. The 100th patient discharged was a 29-year-old mother. Academician Li Lanjuan gave her a "winner" certificate and held her hand warmly, asking about her child's condition. |  |
|  | |  |
|  | |  |
| 1. Disease prevention | No 398: Feb.16, 07:41  【#返岗后如何自我防护#】①人多的办公环境戴口罩；②有症状要积极告知；③#返岗后不要集中就餐#；④乘坐公共交通工具一定要规范戴口罩，有身体不适时不要乘坐公共交通工具；⑤及时做好手卫生。  Literal translation: [#How to protect yourself after returning to work #] ①Wear face masks in a crowded office environment; ②Report if you have symptoms; ③ # Don’t have meals together in a crowded place after returning to work #; ④Must wear face masks when you take public transportation; Do not take public transportation when you are unwell; ⑤ Maintain hand hygiene at all times. |  |
|  | |  |
| 1. Healthcare services | No 351: Feb.12, 06:44  【#火神山医院收治患者超900人#】截至2月11日22时30分，从武汉市定点收治医院转诊而来的确诊患者，陆续抵达火神山医院，患者收治总数达到925人。医院表示，将尽最大努力收治患者。为了加快收治速度，医院坚持边建设、边收治，确保多个病区同时高效收治患者。同时，医院加大了重症患者的收治力度，普通科室也开始适量收治病情较重的患者。目前，重症患者状况基本稳定，部分急危重患者状况有所好转。  Literal translation: 【#HuoShenShan Hospital has accepted more than 900 patients #】 At 22:30 on February 11th, the confirmed patients transferred from the designated hospital in Wuhan were successively transported to HuoShenShan Hospital, accounting for a total number of 925. The hospital said that they would do their best to admit patients. In order to speed up the admission, the hospital will continue to expand to receive and treat patients efficiently. Meanwhile, the hospital has expanded the admission of severely ill patients, and general departments have also started to treat moderately ill patients. Currently, the condition of severely ill patients is basically stable, and the condition of some critically ill patients has improved. |  |
| 1. Uncertainty | No.500: Feb.27, 13:51  【新冠肺炎疫情有可能发源在国外？钟南山：对新冠病毒溯源才能回答这个问题】“疫情不一定发源在中国”是否意味着新冠肺炎的“零号病人”也有可能在国外，通过输入到中国来的？据@广州日报 ，#钟南山补充回应疫情不一定发源在中国#：从科研角度看，“首先发现”和“发源”不能划上等号，但我们也不能就此判断疫情是来自国外。只有对新冠病毒进行溯源，有了结果，才可能回答这个问题。  Literal translation: [COVID-19 may originate from abroad? Zhong Nanshan: Only by tracing the source of COVID-19 can this question be answered] Whether the illustration of “the epidemic does not necessarily originate from China” means that “Patient Zero” of COVID-19 was imported to China? According to @ GuangZhou Daily, #Zhong NanShan added that from the perspective of scientific research, “the first discovery” and “origin from” are not synonymous, but we cannot easily judge that the epidemic originated from abroad. Only by tracing the source of COVID-19 and having the result can this question be answered. |  |
| **Style dimension** |  |  |
|  | |  |
|  | |  |
| 1. Narrative | No.375: Feb.13, 19:41  泪目！#赴汉抗疫护士与儿子隔空对话#：妈，你一定要照顾好自己，不然怎么照顾他们。2月4日，护士长汪凡跟随云南第二批援助湖北医疗队到了抗疫前线。出发前，她怕10岁儿子担心谎称要加班，把他送到姨妈家。直到9日，汪凡才有时间与儿子视频。孩子他爸悄悄录下这段隔空对话。平安回来，早日团聚！  Literal translation: Moved! #Anti-epidemic nurse and her son talking to each other #: “Mom, you must take care of yourself; otherwise how can you take care of patients?” On February 4th, the head nurse Wang Fan followed the second batch of the Yunnan Medical Assistance Team to Hubei as the frontline staff. Before setting off, she was worried about her 10-year-old son, so she lied that she had to work overtime and then put her son in the aunt's house. It wasn't until the 9th that Wang Fan had time to have a video chat with her son. His dad quietly recorded this conversation. Come back safely and reunite soon! |  |
| 1. Non-narrative | No.352: Feb.12, 06:54  扩散周知！#疫情应对10项新政策#，你应该知道①县级以上地方人民政府可依法征用医院周边酒店作为医务人员休息场所；②10类防控物资由政府兜底；③#助学贷款受疫情影响可延期#；④疫情防治临时性工作补助和奖金免征个税；⑤#企业要为职工配发口罩#等防护用品…详戳↓ ​​​​  Literal translation: # Ten new policies you should know# ① The local governments at or above the county level can require hotels around hospitals to serve as a resting place for medical personnel; #; ② The 10 types of prevention materials are funded by the government; ③ # Student loans can be postponed due to the epidemic situation #;  ④ Temporary work subsidies and bonuses issues; ⑤ #Enterprises should distribute masks # and other protective equipment to employees ...... Please see details below |  |
| **Interactive dialogic loop dimension** | |  |
| 1. Links to external sources | No 390: Feb.15, 15:09  【“出舱啦”！#武汉100多名方舱医院患者出院#】2月15日，位于武汉东西湖区的武汉客厅方舱医院，共有100多名新冠肺炎轻症患者经过治疗后集体出院。@人民日报 联动@长江日报 ，听出院的患者都怎么说？戳直播↓*网页链接，人民日报的微博视频*  Literal translation: ["Out of the cabin"! #More than 100 patients from Wuhan square cabin hospital were discharged #] On February 15th, a total of more than 100 patients infected with COVID-19 were discharged collectively after treatment in Wuhan Square Cabin Hospital. @ People’s Daily together with @Chang Jiang Daily - let’s listen to the patients’ feelings and stories. Click live ↓ Web link, Weibo video of People's Daily |  |
| 1. Use of hashtags | No. 385: Feb.14, 22:56  【关注！#新冠肺炎最新数据#：10连降，有的也出现反弹】①#湖北以外新增病例10连降#；②新增疑似病例4连降；③#累计治愈病例超6000例#；④重症率、治愈率和死亡率需要持续关注；⑤部分省份新增确诊病例数量有反弹。  Literal translation: [Attention! #The updated data of COVID-19 #: 10 consecutive drops; some also rebounded] ①# Additional new cases outside Hubei: 10 consecutive drops #; ②New suspected cases: 4 consecutive drops;③# Healed and cured: over 6000 cases #;④The cured rate and mortality rate need continuous attention;⑤The number of confirmed cases in some provinces has rebounded. |  |
| 1. Use of questions to solicit feedback | No 396: Feb.15, 23:37  【共同关注！#14条最新抗疫信息#】鸡鸭会传播病毒？疫苗研究进展如何？病毒传播途径有变化吗？抗疫最新信息↓↓↓扩散周知！ ​​​​  Literal translation: 【Common concern! # the 14 latest anti-epidemic information #] Will chickens and ducks spread the virus? How is the progress of vaccine research? Has the virus transmission route changed? Anti-epidemic latest information ↓↓↓ Let more people see this! |  |
| 1. Use of multimedia | No 257: Feb.5, 10:32  #图解内蒙古住确诊病例楼上被感染病例#近日，“内蒙古达拉特旗新增病例白某某无外出、无接触史，住在确诊病例楼上感染”受到关注。鄂尔多斯发布图解，详述7例确诊患者的人物关系与活动轨迹↓↓ ​​​​  Literal translation: #A picture illustrating a confirmed case about upstairs infection in Inner Mongolia# “Mr. Bai had no history of going out and no contact with confirmed cases but was infected because he was living on the floor above a confirmed patient”. Ordos released a graphic detailing the relationship and activity trajectory of 7 confirmed cases ↓↓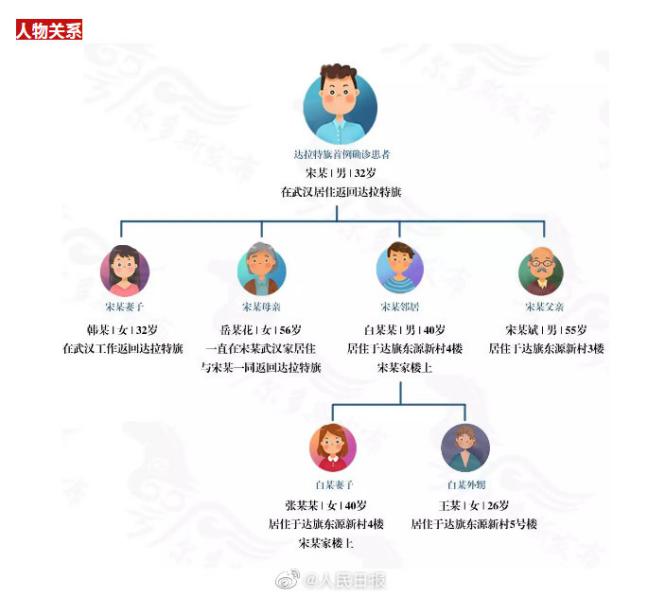 |  |
