# Supplementary material for: Grappling With the COVID-19 Health Crisis: Content Analysis of Communication Strategies and Their Effects on Public Engagement on Social Media
Source: J Med Internet Res. 2020 Aug 24;22(8):e21360. doi: 10.2196/21360 (PMC7446717; doi:10.2196/21360)
Supplement: Multimedia Appendix 2 [file jmir_v22i8e21360_app2.docx]

**Appendix 2: Summary of Inter-rater Reliability Statistics**

| Coding Items | Percent Agreement | Scott's Pi | Cohen's Kappa | Krippendorff's Alpha (nominal) |
| --- | --- | --- | --- | --- |
| Action sentence | 96.7% | 0.948 | 0.948 | 0.949 |
| New evidence sentence | 95.8% | 0.931 | 0.931 | 0.931 |
| Reassurance sentence | 98.3% | 0.851 | 0.851 | 0.852 |
| Disease prevention sentence | 98.3% | 0.883 | 0.883 | 0.884 |
| Healthcare services sentence | 96.7% | 0.881 | 0.881 | 0.881 |
| Uncertainty sentence | 100% | 1 | 1 | 1 |
| Content (dominant category) | 100% | 1 | 1 | 1 |
| Narrative sentence | 95% | 0.851 | 0.851 | 0.851 |
| Non-narrative sentence | 95% | 0.942 | 0.942 | 0.942 |
| Style (dominant category) | 100% | 1 | 1 | 1 |
| Links to external sources-count | 97.5% | 0.843 | 0.844 | 0.844 |
| Use of hashtags-count | 100% | 1 | 1 | 1 |
| Use questions to solicit feedback-count | 100% | 1 | 1 | 1 |
| Use of multimedia-count | 95.8% | 0.857 | 0.857 | 0.858 |
| Interactive (dominant category) | 100% | 1 | 1 | 1 |
| Share | 100% | 1 | 1 | 1 |
| Comment | 100% | 1 | 1 | 1 |
| Like | 100% | 1 | 1 | 1 |
